# Ureteral obstruction and hydronephrosis caused by foreign body: A case report and literature review

**DOI:** 10.1097/MD.0000000000017780

**Published:** 2019-11-01

**Authors:** Zhe-Wei Zhao, Xing-Cheng Wu, Jian-Hua Deng, Peng-Hu Lian, Xue-Bin Zhang

**Affiliations:** Department of Urology, Peking Union Medical College Hospital, Chinese Academy of Medical Science and Peking Union Medical College, Beijing, China.

**Keywords:** foreign body, hydronephrosis, ureteral obstruction

## Abstract

**Rationale:**

Foreign bodies related ureteral obstruction and hydronephrosis is rare and usually cause numerous problems for clinical physicians.

**Patient concerns:**

We report a 36-year-old female who was referred to our hospital due to a 4-year history of dull pain on the left back.

**Diagnosis:**

X-ray and abdominal CT revealed a foreign body around the upper part of the left ureter with ureteral obstruction and hydronephrosis.

**Interventions:**

Laparoscopy was performed and a 3-cm sewing needle was removed successfully.

**Outcomes:**

After 6 months’ follow-up, the patient's ureteral obstruction and hydronephrosis were significantly reduced, and the double-J ureteral stent was removed.

**Lessons:**

This case indicated that ureteral obstruction and hydronephrosis caused by foreign bodies needed to be early diagnosed and located. Invasive therapies rather than conservative treatments are preferred to remove the FBs and relieve obstruction.

## 1 Introduction

Foreign body (FB) is a common surgical emergency. Compared with ingestion and iatrogenic injury, percutaneous insertion is a relative rare entrance of FB. The common type of percutaneous insertion FB is sewing needles and most patients are children. Injury caused by sewing needles usually occur accidentally except for some child abuse or self-inflicted injuries caused by suicide attempt.^[1,2]^ Migration of FBs is the main pathogenesis, which can lead to abscess formation, inflammation, or direct invasion.

FB attached to the genitourinary tract often causes many concerns for clinicians, including hematuria, infection, hydronephrosis, and renal failure. Imaging studies are sometimes insufficient for detecting FB. To prevent serious complications, an accurate diagnosis and effective FB extraction are particularly important. Here, we report a rare case of ureteral obstruction and hydronephrosis caused by retroperitoneal FB and present a review of the related literature.

## 2 Case report

**Figure 1:**
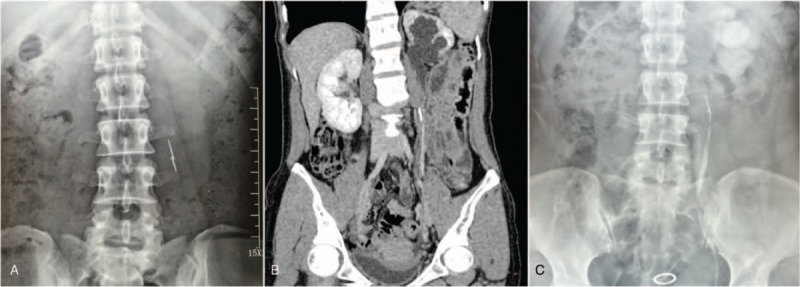
(A) KUB showed a foreign body on the left side of the spine early in 2014. (B) Computed tomography (CT) scan revealed moderate hydronephrosis of the left kidney. (C) Retrograde pyelogram showed stricture of the upper ureter in the region near the foreign body.

**Figure 2:**
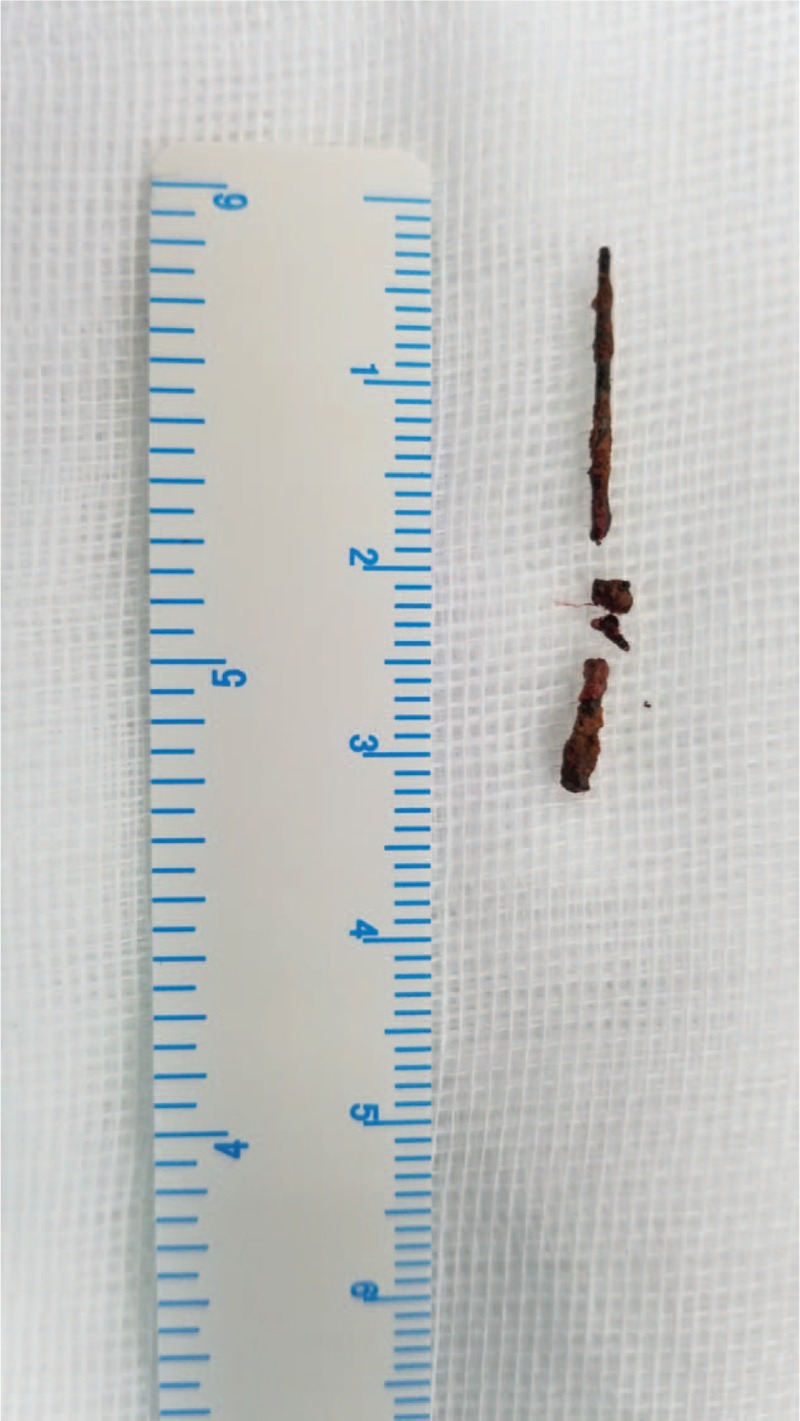
Photograph of the foreign body. It was identified as a 3 cm sewing needle.

A 36-year-old female presented to the hospital with a 4-year history of dull pain on the left back and a foreign body on the left side of the spine. She denied hematuria, dysuria, or frequency. Early in 2014, an X-ray of the kidney, ureter, and bladder (KUB) showed a foreign body on the left side of the spine (Fig. 1A). Since then, she was asymptomatic and did not receive any treatment. The patient denied ingestion of any FBs and her past medical and surgical history was also unremarkable. The history was carefully re-taken, and her parents recalled that a needle-like FB may have been accidentally inserted into her body percutaneously when she was a child. After admitting her to our hospital, a computed tomography (CT) scan of the abdomen and pelvis revealed moderate hydronephrosis of the left kidney (Fig. 1B). A linear high-density foreign body was found around the upper part of the left ureter. The renal blood flow demonstrated a poor perfusion and function of left kidney with eGFR 16.80 ml/(min·1.73 m^2^). A retrograde pyelogram showed stricture of the upper ureter (Fig. 1C) in the region near the foreign body. A double-J ureteral stent was placed to relieve the obstruction. Laparoscopy was performed, and a 3-cm sewing needle (Fig. 2) was removed successfully. After 6 months’ follow-up, the patient's ureteral obstruction and hydronephrosis were significantly reduced, and the double-J ureteral stent was removed.

Patients have provided informed consent for publication of the case.

## 3 Discussion

FBs are common urologic problems and most of them are inserted into the genitourinary tract.^[3]^ However, migration from adjacent organs, although rare, can also cause urologic problems when externally attached to the genitourinary tract. It often has an acute onset and rapid progression, accompanied by various clinical symptoms. However, it may be difficult to diagnose when late complications occur, such as hydronephrosis and renal failure.

To the best of our knowledge, only 12 cases of FB-induced hydronephrosis have been reported in English in MEDLINE (Table 1).^[4–14]^ The most common pathogenesis of these cases is ingestion causing perforation.^[6,8–10,12–14]^ Migration of perforation by FBs can involve adjacent structures, such as the ureter in this case, which induces obstruction because of abscess formation, inflammation, or direct invasion. Only three cases of FBs in the bladder have been reported,^[4,5,10]^ since most patients with bladder FBs undergo emergency intervention. FBs in the urinary bladder can cause obstruction and hydronephrosis directly by the FB itself, and also secondarily by stones. To our knowledge, this is the first reported case of this urologic complication caused by FB migration after percutaneous insertion. This case also serves to alert parents to the safe storage of sharp objects while taking care of children. The types of these FBs include needles, toothpicks, pins, brush bristle, fish bone, bullets, and silicon products. Most of them are long, stiff, and sharp objects with high perforation and migration risks.

**Table 1:**
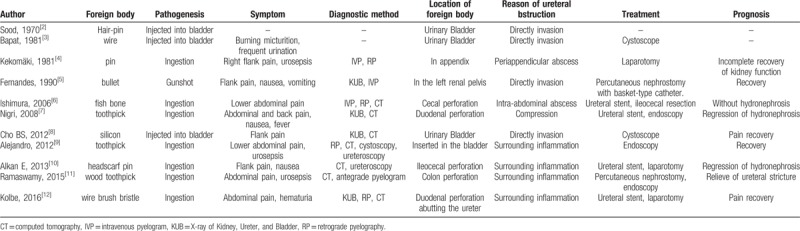
Cases of foreign body causing ureteral obstruction and hydronephrosis.

The various positions of FBs may present different clinical features. FBs in the bladder can lead to dysuria, frequent micturition, urgency of urination and urinary pain. Patients with gastrointestinal (GI) perforation due to FBs usually present with abdominal pain and gastrointestinal bleeding.^[9]^ However, a number of patients are asymptomatic until they develop late complications. The common symptoms of FBs caused hydronephrosis are flank pain, nausea, hematuria, and urosepsis.

Early diagnosis and treatment are essential for avoiding late complications. X-rays and CT scans are most commonly used examinations. Depending on the properties of the FBs, sometimes CT scan is necessary when X-ray is unremarkable.^[14]^ Intravenous pyelogram and retrograde pyelogram are performed to evaluate obstruction of the urinary system. Cystoscopy or ureteroscopy are alternative choices when all the above methods are negative.^[11]^

Invasive therapies rather than conservative treatments are preferred to remove the FBs and relieve obstruction. Cystoscope is an effective method to resolve the obstruction caused by FBs in the bladder. As for FBs from the GI tract, endoscopy is usually the first choice after locating the site of perforation. If this is unsuccessful, laparoscopy or laparotomy with or without fluoroscopic guidance should be carried out. A double-J ureteral stent or percutaneous nephrostomy tube was used to relieve the obstruction in some cases and was removed after about 1 month.^[9,12,13]^ However, early removal of ureteral stent may lead to recurrent hydronephrosis.^[8]^

Most patients are asymptomatic and have good recovery from hydronephrosis after follow-up. Only one case reported incomplete ultimate remission of kidney function because of the serious nature of the obstruction.^[6]^

In conclusion, FB migration causing ureteral obstruction and hydronephrosis is rare. These cases are usually caused by ingestion and sexual motives. The patients may present with different clinical features according to the various position of FBs. Early and accurate diagnosis and treatment are essential to avoid late complications. The patients usually have good prognoses with recovery from hydronephrosis.

## Acknowledgments

We would like to thank Editage [www.editage.cn] for English language editing. The authors report no conflict of interest.

## Author contributions

**Conceptualization:** Zhe-Wei Zhao, Xue-Bin Zhang.

**Data curation:** Jian-Hua Deng, Peng-Hu Lian.

**Methodology:** Xing-Cheng Wu.

**Supervision:** Xue-Bin Zhang.

**Visualization:** Jian-Hua Deng, Peng-Hu Lian.

**Writing – original draft:** Zhe-Wei Zhao, Xing-Cheng Wu.

**Writing – review & editing:** Xue-Bin Zhang.
